# Baicalin Mitigates *Pasteurella multocida*-Induced Pulmonary and Vascular Injury via NLRP3/COX-2 Inhibition in Mice

**DOI:** 10.3390/ani15203055

**Published:** 2025-10-21

**Authors:** Dan Zhang, Chengzhuo Zhao, Yunda Xue, Qirong Lu, Yu Liu, Jianglin Xiong, Chun Ye, Shulin Fu, Zhongyuan Wu, Yinsheng Qiu, Pu Guo

**Affiliations:** 1 Hubei Key Laboratory of Animal Nutrition and Feed Science, School of Animal Science and Nutritional Engineering, Wuhan Polytechnic University, Wuhan 430023, China; zhangd202509@126.com (D.Z.); 15271771535@163.com (C.Z.); 15823132897@163.com (Y.X.); qirongluvet@whpu.edu.cn (Q.L.); lyywfy@foxmail.com (Y.L.); xiongjianglin@126.com (J.X.); yechun@whpu.edu.cn (C.Y.); shulinfu@whpu.edu.cn (S.F.); zhongywu@whpu.edu.cn (Z.W.); 2 Wuhan Engineering and Technology Research Center of Animal Disease-Resistant Nutrition, School of Animal Science and Nutritional Engineering, Wuhan Polytechnic University, Wuhan 430023, China

**Keywords:** baicalin, inflammatory response, *Pasteurella multocida*, vascular injury, pneumonia, mouse model, inflammasome

## Abstract

**Simple Summary:**

*Pasteurella multocida* is a bacterium that can spread from animals to humans and is a major cause of lung infections in farm animals. This infection often leads to severe hemorrhagic pneumonia and harmful inflammation that damages both the lungs and blood vessels, posing a risk to both animal health and public safety. This study investigated whether a natural compound called baicalin could protect against this inflammation. Using a mouse model of infection, we found that baicalin treatment significantly reduced weight loss and improved damage in lung and blood vessel tissues. It worked by lowering the levels of key inflammation-related proteins, specifically NLRP3, COX-2, IL-1β, and IL-18. Our results indicate that baicalin protects the lungs and blood vessels by blocking the activation of these inflammatory pathways. Therefore, baicalin shows promise as a potential treatment for combating the severe inflammatory injury caused by this bacterial infection, which could be valuable for improving outcomes in both veterinary and public health.

**Abstract:**

*Pasteurella multocida* (*P. multocida*), a zoonotic bacterium, is one of the most common respiratory pathogens in animal husbandry and causes many public health problems. Infection by *P. multocida* can cause hemorrhagic pneumonia and induce pulmonary and even vascular inflammatory injury. Baicalin has protective and/or therapeutic effects in a variety of lung diseases. However, whether it also protects against vascular inflammatory injury caused by *P. multocida* infection in vivo remains to be investigated. The present study used mice infected with *P. multocida* as a model to explore the alleviation of pulmonary and vascular inflammatory injury by baicalin. Baicalin significantly reduced weight loss, improved the pathological changes of lung and blood vessels, and reduced the expression of the inflammation-related proteins NLRP3, COX-2, IL-1β, and IL-18 in lung and blood vessel tissues. The signal inhibition of NLRP3 and COX-2 may be a key therapeutic pathway to treat *P. multocida*-induced pulmonary and vascular inflammatory injury. These findings suggest that baicalin inhibits the activation of inflammation to protect pulmonary and vascular injury in vivo. Hence, baicalin exhibits therapeutic potential in the treatment of pulmonary and vascular injury.

## 1. Introduction

*Pasteurella multocida* (*P. multocida*), a zoonotic bacterium, is one of the most common respiratory pathogens in animal husbandry, infecting swine, bovine, goats, and poultry, resulting in high morbidity and mortality as well as substantial economic losses. An occurrence of 9.4% of chickens, ducks, quails, and turkeys infected with *P. multocida* was reported in Egypt [1], and 27.2%, 31.6%, and ~20% of pigs in India, the USA, and China, respectively, were infected [2,3,4]. In Africa, 85.4% and 9.0% of cattle and sheep, respectively, had *P. multocida*, as well as 67.2% of cattle in Asia, 100% of cattle in the USA and Europe [5], 93.8% of cattle in Ethiopia [6], 20% of poultry/rabbits in Egypt [7], and 7.57% of cattle/buffaloes in Pakistani [8], which has caused huge economic losses to the livestock industry. Although the prevalence varies in different regions and species, it can cause similar respiratory symptoms, pneumonia, hemorrhagic septicemia, and even death [9,10,11]. Increased pulmonary endothelial inflammation, vascular leakage, and leukocyte infiltration are key features in hemorrhagic septicemia [12], which indicates that hemorrhagic septicemia may be accompanied by necrosis and injury to the blood vessel walls [13], such that infection of *P. multocida* may induce pulmonary and even vascular inflammatory injury. *P. multocida* can infect humans through wounds or the respiratory tract, causing diseases, such as pneumonia, sepsis, and meningitis [14,15], which make *P. multocida* infections a public health problem.

A critical and escalating concern is the widespread antimicrobial resistance. High resistance rates of *P. multocida* are commonly observed against ampicillin (41.1%), tetracycline (38.2%), trimethoprim/sulfamethoxazole (70.1%), and erythromycin (67.5%) [8,16]. Due to its devastating prognosis combined with the high cost of treatments and vaccines needed for treatment and prevention, *P. multocida* is considered a significant pathogen that requires more collaborative research [17,18]. Chinese herbal medicine has received increasing attention for the treatment and prevention of bacterial diseases in animals [19,20]. Baicalin is a flavonoid extracted from the roots of *Scutellaria baicalensis* (Lamiaceae), which has a wide range of biological activities, including anti-cancer, anti-oxidation, anti-inflammatory, antiviral, and antibacterial [21,22,23,24]. Thus, its wide application in medicine, health care, food safety, and other fields has promoted it to become a focus of global research and development in recent years [25,26].

The application of baicalin in the treatment of acute lung injury (ALI), chronic obstructive pulmonary disease, lung fibrosis, and other lung diseases is also being widely explored [27,28]. Intraperitoneal injections of 25, 50, and 100 mg/kg b.w. of baicalin for 12 h significantly improved the pathological changes in the lung, reduced pulmonary edema, and decreased the cytokine (TNF-α, IL-1β, and IL-6) levels in LPS-induced ALI mice [29]. Also, 50, 100, and 200 mg/kg b.w. of baicalin administered orally for 7 days exerted anti-inflammatory effects by inhibiting the CX3CL1-CX3CR1 axis and NF-κB activation in mice [30]. Furthermore, 182, 364, and 728 mg/kg b.w. of baicalin reduced the production of TNF-α, IL-1β, IL-6, and IL-10 by the down-regulation of TLR4 and the inhibition of NF-κB after 5 days of oral treatment in rats with acute pneumonia caused by multidrug-resistant Pseudomonas aeruginosa [31]. These findings indicate that baicalin has protective and/or therapeutic effects in a variety of lung diseases. However, whether it also protects against vascular inflammatory injury caused by *P. multocida* infection in vivo remains to be investigated.

The mouse model is extensively utilized as an in vivo tool to mimic the infection processes observed in humans and animals. In vivo studies in mice demonstrated that *P. multocida* infection increases BBB permeability, consistent with findings in human cell models [14]. Moreover, mouse models validated that *P. multocida* infection causes lung damage and bacteremia [32]. Furthermore, the mouse model was used to investigate the protective effects of 18β-glycyrrhetinic acid (GA) against *P. multocida*-induced vascular inflammatory injury [33]. Thus, this study aimed to use a mouse model to study the therapeutic effect of baicalin on *P. multocida* and provide a research direction for the development of anti-*P. multocida* drugs, thus laying the foundation for reducing the economic loss of animal husbandry and providing treatment or preventive drugs for reducing the disease of people engaged in related industries.

## 2. Materials and Methods

### 2.1. Reagents and Chemicals

Baicalin (CAS NO. 21967-41-9) was purchased from Biopurify Phytochemicals Ltd. (Chengdu, China), and dimethyl sulfoxide (DMSO) was purchased from Sigma-Aldrich (Saint-Quentin-Fallavier, France).

### 2.2. Bacteria and Culture Conditions

*P. multocida* strain HB03 was provided by Professor Bin Wu and Dr. Peng Zhong of Huazhong Agricultural University (Wuhan, China). The strain was cultivated in tryptic soy broth (Difco Laboratories, Franklin Lakes, NY, USA) or on tryptic soy agar (TSA) (Difco Laboratories, Franklin Lakes, NY, USA) with 5% fetal bovine serum (Tianhang, Hangzhou, China) at 37 °C.

### 2.3. Animals

BALB/c female mice 4–5 weeks old from the Centre of Laboratory Animals of Hubei Province, Wuhan, China, were used for this study. The study was approved by the Animal Care and Use Committee (Wuhan Polytechnic University, Wuhan, China, WPU202310008).

### 2.4. Experimental Design

The experiments were designed according to a previously published paper [33]. Fifty mice were randomly divided into five groups with ten mice in each group. The groups included uninfected mice (control group), untreated mice infected with *P. multocida* (model group), and *P. multocida*-infected mice treated with 25 mg/kg b.w. of baicalin (baicalin 25 mg/kg b.w. group), *P. multocida*-infected mice treated with 50 mg/kg b.w. of baicalin (baicalin 50 mg/kg b.w. group), and *P. multocida*-infected mice treated with 100 mg/kg b.w. of baicalin (baicalin 100 mg/kg b.w. group). Before injection, the baicalin was dissolved in DMSO and diluted in phosphate-buffered saline (PBS).

**Figure 1:**
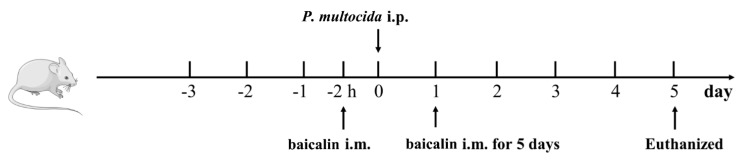
Schematic design of the study.

The day before treatment, all animals received fresh water only (no basic food) (Figure 1). On day 1, the model and baicalin groups were injected i.p. with 0.1 mL of baicalin solution (*P. multocida* at a dose of 800 CFU/mL), while the control group was injected i.p. with 0.1 mL of PBS. Six hours later, baicalin groups were injected i.m. with different concentrations of baicalin, while the control and model group mice were intramuscularly injected with 0.1 mL of the same dose of PBS. The daily injections occurred over 5 consecutive days. Mice were then sacrificed, and lung tissue and abdominal aorta vessels were collected for subsequent experiments. This experiment was instigated in strict accordance with the recommendations of the China Regulations for the Administration of Affairs Concerning Experimental Animals 1988 and the Hubei Regulations for the Administration of Affairs Concerning Experimental Animals 2005.

### 2.5. Histopathological Analysis

All histopathological tests were performed using standard laboratory procedures. The tissue slices were obtained by fixation, embedding, and dyeing the lung tissue and abdominal aortic vessels, and the specific operation is found in our previous research [34]. Morphological alterations in each group were observed under a microscope.

### 2.6. Immunohistochemical Assays for IL-1β, IL-18, COX-2, and NLRP3

Immunohistochemical assays of lung tissue and abdominal aortic vessels were performed as previously reported [35]. The tissue slices were deparaffinized, antigenically repaired, incubated with primary protein antibody and secondary antibody, analyzed by DAB chromatography, re-stained, and sealed. Stained proteins were observed under a microscope. COX-2 (12375-1-AP), IL-18 (10663-1-AP), IL-1β (16806-1-AP), and NLRP3 (27458-1-AP) polyclonal antibody were used.

### 2.7. Molecular Docking

The AlphaFold3 (AF3) structure prediction platform was used for structure modeling and docking calculations. The amino acid sequences of COX-2 and mouse NLRP3 proteins from UniProt (https://www.uniprot.org/, accessed on 15 August 2025) and the Simplified Molecular Input Line Entry Specification (SMILES) molecular formula information of baicalin from PubChem (https://pubchem.ncbi.nlm.nih.gov, accessed on 15 August 2025) were obtained. Three-dimensional structure models of the COX-2–baicalin and NLRP3–baicalin complexes were constructed by utilizing the protein–molecule complex prediction function of AF3. During the prediction process, the complete protein sequence and the SMILES structure of baicalin were input, and the AF3 multi-chain composite modeling mode was enabled. The conformation of the complex was predicted independently multiple times, and the result with the highest confidence was selected for subsequent analysis. The acquired complex structure was visualized in three dimensions, and the binding site was analyzed using ChimeraX 1.8, by which the spatial conformation of the protein–ligand complex was displayed, the surface potential was rendered, and key amino acid residues were labeled. This provided an intuitive visualization of the interaction patterns of baicalin with the binding pockets of COX-2 and NLRP3.

### 2.8. Molecular Dynamics Simulations and MMPBSA

Docking-derived poses of the baicalin–COX-2 and baicalin–NLRP3 complexes were used as starting structures. Explicit solvent MD simulations were performed in GROMACS using the amber99sb protein force field and TIP3P water; baicalin was parameterized with GAFF2 (generated with Antechamber and converted to GROMACS via ACPYPE). Systems were solvated in a triclinic box (≥1.0 nm buffer), neutralized with counter-ions, and set to 0.15 M NaCl. After Steepest Descent minimization, NVT (300 K, 0.5 ns, v-rescale) and NPT equilibration (300 K, 1 bar, 1.0 ns, Parrinello–Rahman) were carried out with heavy-atom positional restraints. Production runs were 100 ns (2 fs timestep; LINCS for bonds to hydrogens; 1.0 nm LJ/Coulomb cutoffs; PME for long-range electrostatics; neighbor list updated every 20 steps; frames saved every 10 ps). Complex backbone RMSD was monitored to identify a plateau (RMSD-stable) window for energy evaluation; frames were uniformly sampled from this segment (typically every 50–100 ps) and MM-PBSA-binding free energies were computed with gmx-MMPBSA using the single-trajectory protocol. Polar solvation was treated with PB/GB and the nonpolar term with SASA/LCPO; configurational entropy was not explicitly included, so the reported ΔG values are estimates used for relative affinity comparison.

### 2.9. Statistics

Statistical analysis was performed using SPSS 18.0 for Windows. All results were presented as mean ± SD. One-way ANOVA followed by Tukey HSD post hoc tests was used to analyze group differences. * = *p*  <  0.05 vs. control group; ** = *p*  <  0.01 vs. control group; # = *p*  <  0.05 vs. model group; ## = *p*  <  0.01 vs. model group.

## 3. Results

### 3.1. Altered Mental Status

**Figure 2:**
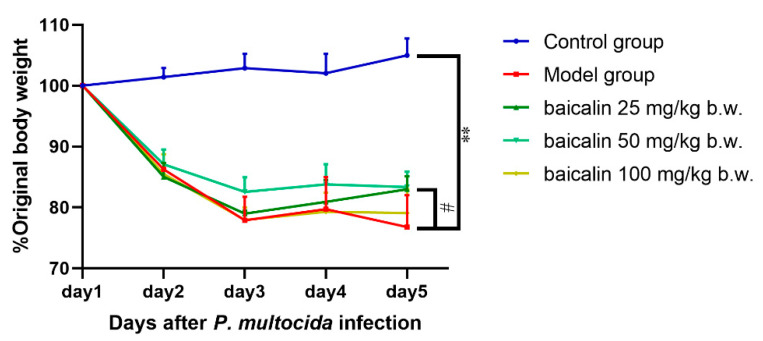
Body weight changes in *P. multocida*-infected mice treated with baicalin. ** = *p*  <  0.05 vs. control group; # = *p*  <  0.05 vs. model group.

Mice in the control group exhibited good mental states, ate normally, had smooth coats, and their body weights showed an upward trend (Figure 2). Mice infected by *P. multocida* were listless, curled up, anorexic, and depressed with messy fur, secretions in the corners of the eyes, and adhesion of feces, and their body weight gain was less than that in the control group. However, the mental states of mice in the baicalin treatment groups were significantly better than those of mice in the model group, and the secretions in the corner of the eye and fecal adhesion were reduced. Weight gain was observed in the baicalin treatment groups. Interestingly, weight regain was highest in the 50 mg/kg b.w. baicalin group, the medium-dose group.

### 3.2. Histological Evaluation

**Figure 3:**
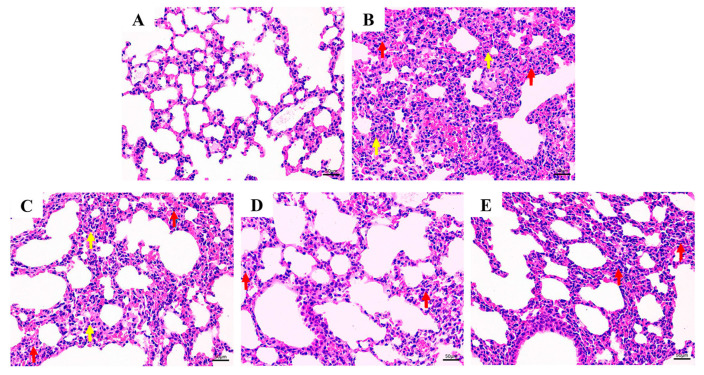
Histopathological evidence of the effect of baicalin on mouse lung tissue damaged by infection with *P. multocida* (scale bar = 50 μm). (**A**) Control group without infection. (**B**) Model group infected but untreated. (**C**) Baicalin 25 mg/kg b.w. group (arrow indicates significant bleeding in the pyramidal cell layer). (**D**) Baicalin 50 mg/kg b.w. group. (**E**) Baicalin 100 mg/kg b.w. group (red arrow: alveolar injury; yellow arrow: inflammatory cells in the tissue).

Significant histopathological changes in the lung tissue were observed in the *P. multocida*-infected groups (Figure 3). The histopathological tests showed a basically normal lung tissue structure, clear alveolar contours, clear boundaries, and no edema or infiltration of inflammatory cells in the control group mice (Figure 3A). The lung tissue structure of the model group mice was severely abnormal, with disordered arrangement of alveoli, blurred boundaries of each alveoli, severe parenchyma of alveoli, proliferation of a large number of alveolar epithelial cells (Figure 3B, red arrow), alveolar atrophy accompanied by thickening of alveolar walls, congestion and expansion of capillaries in the alveoli, and infiltration of inflammatory cells in the tissue (Figure 3B, yellow arrow). In the low-dose treatment group with baicalin, the lung tissue structure was moderately abnormal, with a disordered arrangement of alveoli in the field of vision, blurred boundaries of each alveolus (Figure 3C, red arrow), and a small amount of inflammatory cell infiltration in the tissue (Figure 3C, yellow arrow). In the medium-dose treatment group of baicalin, the lung tissue structure was slightly abnormal, with some alveolar epithelial cells proliferating and bronchial epithelial cells arranged tightly in a regular manner without significant shedding. No obvious infiltration of inflammatory cells was observed in the organization (Figure 3D). Moreover, the high-dose treatment group with baicalin showed mild abnormalities in lung tissue structure, with some alveolar epithelial cells proliferating and bronchial epithelial cells arranged tightly in a regular manner without significant shedding. No obvious infiltration of inflammatory cells was observed in the organization (Figure 3E).

**Figure 4:**
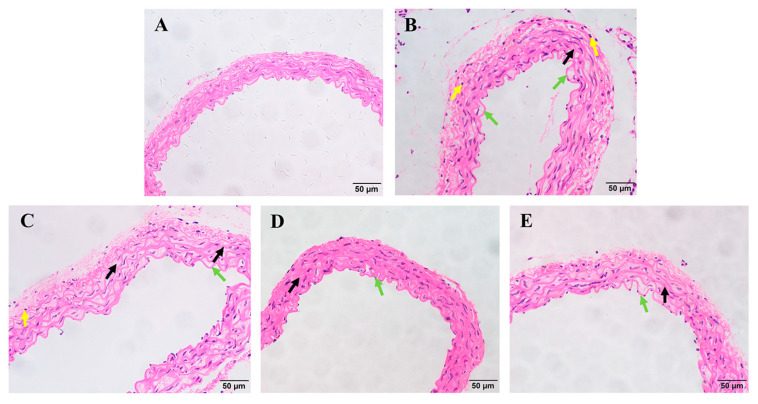
Histopathological evidence of the effect of baicalin on mouse blood vessel tissue infected with *P. multocida* (scale bar = 50 μm). (**A**) Control group without infection. (**B**) Model group infected but untreated. (**C**) Baicalin 25 mg/kg b.w. group (arrow indicates significant bleeding in the pyramidal cell layer). (**D**) Baicalin 50 mg/kg b.w. group. (**E**) Baicalin 100 mg/kg b.w. group (black arrow: the vascular structure was moderately abnormal, and some elastic fibers had unclear structure, disordered arrangement, and local fracture; yellow arrow: inflammatory cells; green arrow: partial shedding of endothelial cells in the intima of the vessel).

Significant histopathological changes in the blood vessels were observed in the *P. multocida* infection groups (Figure 4). The results of histopathological tests showed basically normal blood vessel tissue structure, clear elastic fibers, normal structure of endothelial cells, and no obvious inflammatory cell infiltration in the tissues in the control group mice (Figure 4A). In the model group, the vascular structure was moderately abnormal, and some elastic fibers had unclear structure, disordered arrangement, and local fracture (Figure 4B, black arrows). Endothelial cells were structurally normal and partially exfoliated (Figure 4B, green arrow), and many inflammatory cells had infiltrated the tissue (Figure 4B, yellow arrows). In the low-dose baicalin treatment group, the vascular structure was slightly abnormal, and the elastic fiber structure in the visual field was not clear, with local breakage (Figure 4C, black arrow); partial shedding of endothelial cells in the intima of the vessel was evident (Figure 4C, green arrow), and inflammatory cells infiltrated the tissue (Figure 4C, yellow arrows). In the medium-dose and high-dose treatment groups, there were mild abnormalities in the vascular structure, with unclear elastic fiber structure within the visual field and local rupture (Figure 4D,E, black arrow). No obvious detachment of endothelial cells in the vascular intima was observed (Figure 4D,E, green arrow), and no obvious inflammatory cell infiltration was found in the vascular adventitia.

### 3.3. Expression of IL-1β, IL-18, COX-2, and NLRP3

**Figure 5:**
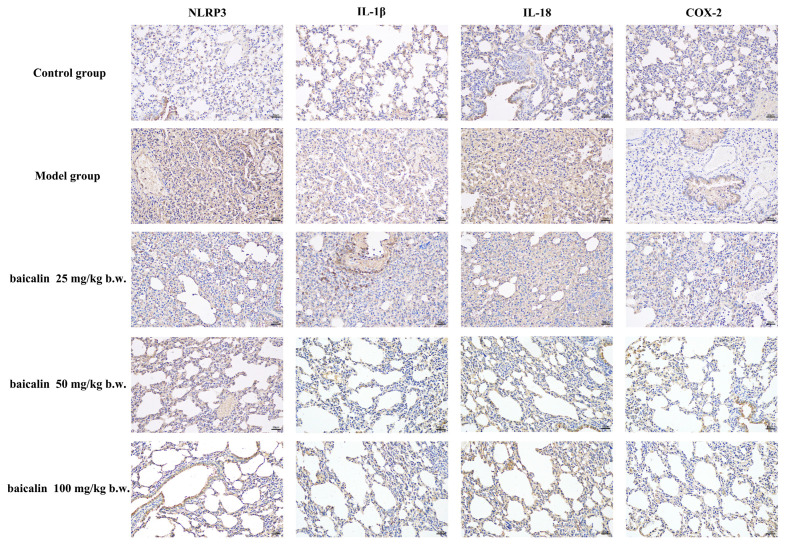
Immunohistochemical effects of baicalin on the lung tissue of mice infected with *P. multocida* (scale bar = 50 μm).

**Figure 6:**
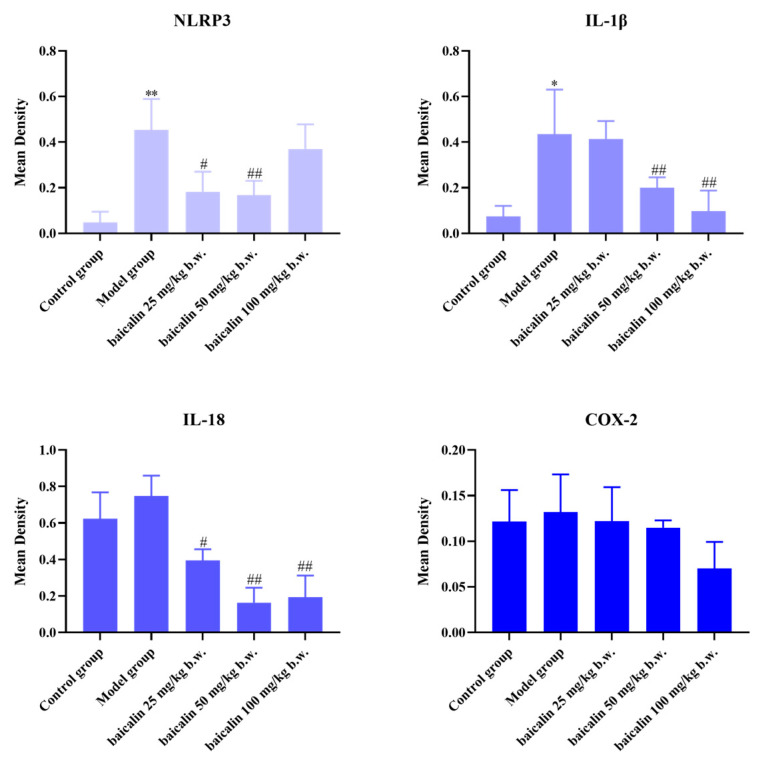
Quantitative IHC analysis of lung tissues (* = *p*  <  0.05 vs. control group; ** = *p*  <  0.01 vs. control group; # = *p*  <  0.05 vs. model group; ## = *p*  <  0.01 vs. model group.).

**Figure 7:**
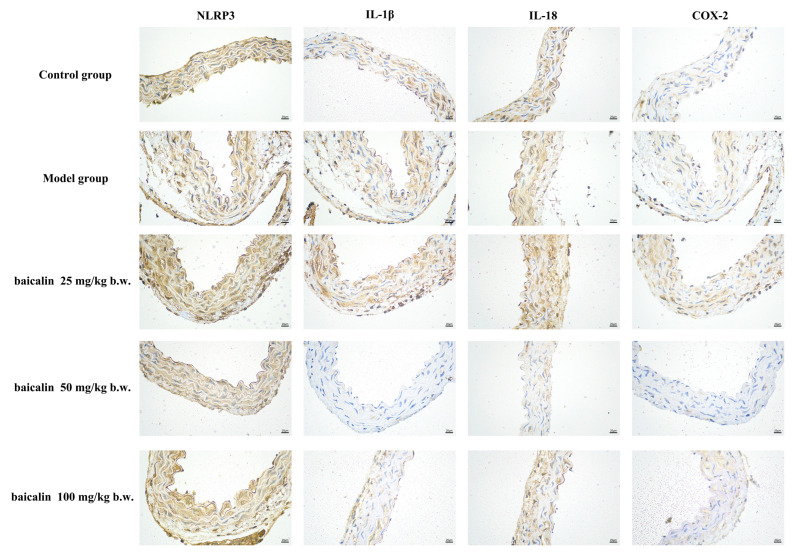
Immunohistochemical effects of baicalin on the blood vessels of mice infected with *P. multocida* (scale bar = 20 μm).

**Figure 8:**
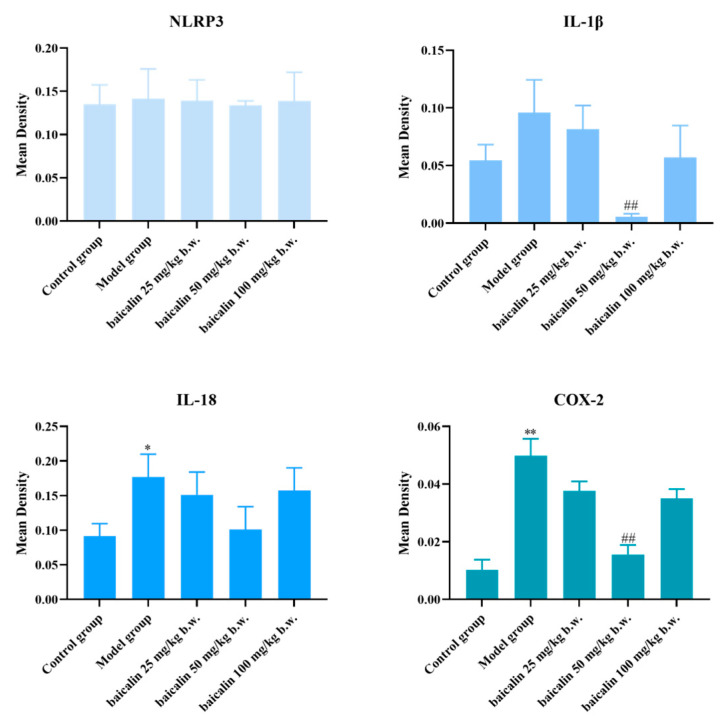
Quantitative IHC analysis of vascular tissues (* = *p*  <  0.05 vs. control group; ** = *p*  <  0.01 vs. control group; # = *p*  <  0.05 vs. model group; ## = *p*  <  0.01 vs. model group.).

NLRP3, IL18, IL-1β, and COX-2 showed positive expression in the brain or blood vessels of mice in all groups (Figure 5, Figure 6, Figure 7 and Figure 8). The expression of these inflammation-related proteins was significantly increased (*p* < 0.01) in the mice infected with *P. multocida*. NLRP3, IL18, IL-1β, and COX2 showed slightly negative expression in the BA-treated groups compared with *P. multocida*. The expression of these four inflammation-related proteins was higher after infection with *P. multocida*. NLRP3, IL18, IL-1β, and COX-2 showed slightly less expression in the baicalin treatment groups compared to the model.

### 3.4. Analysis of Molecular Docking Results

**Figure 9:**
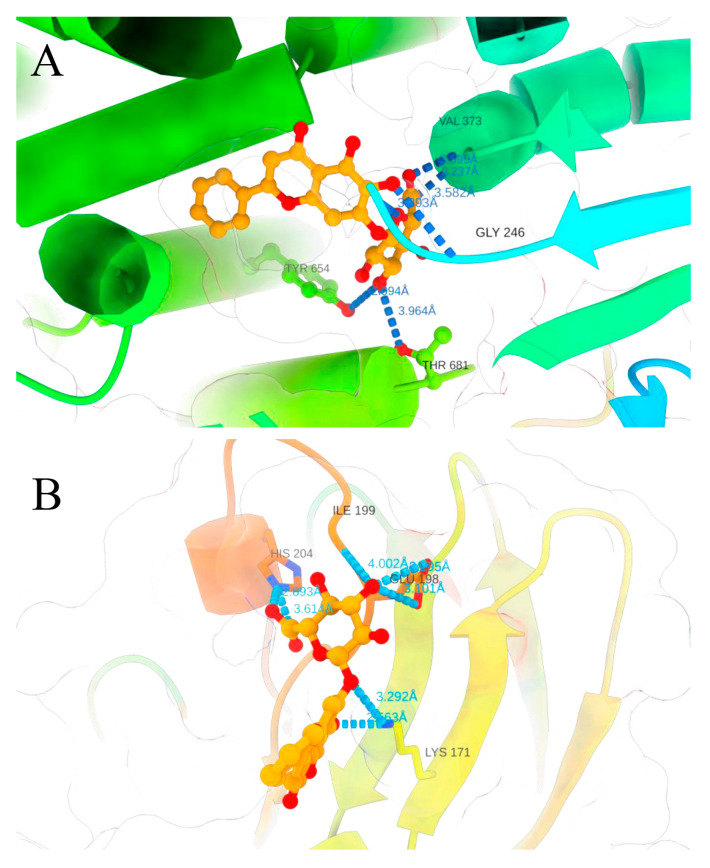
Molecular docking diagram by AlphaFold3. Molecular 3D models of the binding of baicalin with mouse NLRP3 and COX-2: (**A**) baicalin–NLRP3 (ranking_score = 0.7179); (**B**) baicalin–COX-2 (ranking_score = 0.7064).

Baicalin can bind the docking pocket of each target protein with good docking activity (Figure 9). Baicalin binds to NLRP3 by forming hydrogen bonds with GLY-246, VAL-373, and THR-681 (ranking_score = 0.7179, Figure 9A). Similarly, quercetin is predicted to dock in the binding pocket of COX2 via hydrogen bonds with ILE-199, LYS-171, and HIS-204 (ranking_score = 0.7064, Figure 9B). To provide quantitatively interpretable results with clear physical meaning, we performed 100 ns explicit solvent molecular dynamics simulations (amber99sb) for the Baicalin–COX-2 and Baicalin–NLRP3 complexes and estimated binding free energies using gmx-MMPBSA over the RMSD-stable portion of the trajectories. The results indicate ΔG values of approximately −42 kcal/mol for COX-2 and −38 kcal/mol for NLRP3. Baicalin may affect its function by competitively inhibiting the binding of the docking pocket to the target receptor. These interactions might play key roles in alleviating *P. multocida* infection.

## 4. Discussion

As the most common respiratory pathogen in animal husbandry, pneumonia caused by *P. multocida* results in significant economic losses for producers. Necrosuppurative/necrohemorrhagic fibrinous pleuropneumonia, which is characterized by multifocal areas of coagulation necrosis of the lung parenchyma and associated with suppurative inflammatory exudation, necrosis of the vessel walls, and hemorrhagic multifocal areas, is frequent with *P. multocida* infection [13]. Therefore, infection by *P. multocida* may induce pulmonary inflammatory injury and even vascular inflammatory injury. Poor diagnosis and high costs of drugs and vaccines for treatment and prevention make *P. multocida* an important public health threat that requires more collaborative efforts to address [17]. Conventional antibiotic treatment cannot solve the problem of vascular damage caused by *P. multocida* [33]. Natural products may become candidate drugs to alleviate pulmonary and vascular inflammatory injury [36]. Thus, exploring the pulmonary and vascular inflammatory injury caused by *P. multocida* infection and the regulatory role of natural products has important clinical significance. In our study, mice infected with *P. multocida* were used as a model to explore the alleviation of pulmonary and vascular inflammatory injury by baicalin. Baicalin significantly improved the pathological changes of the lung and blood vessels and reduced the expression of related inflammatory proteins caused by *P. multocida* infection in mice. This study elucidated the regulatory role of baicalin in pulmonary and vascular inflammatory injury, providing a theoretical basis for the prevention and control of *P. multocida* infection, as well as the development of alternative antibiotic treatments and feed additives.

Previous studies and our study indicate that mice are an effective model for studying *P. multocida* infection, with the main symptoms of listlessness, dull and messy fur, anorexia, depression, reluctance to move, and congestion and hemorrhage in the lungs [33,37]. Moreover, our study displays that the body weight of mice in *P. multocida*-infected mice significantly decreased compared with uninfected mice, further illustrating the usefulness of a mouse model to study *P. multocida* infection, which is consistent with the fact that the *P. multocida* HB03 is classified as hypervirulent. Furthermore, mice infected with *P. multocida* exhibited significant pulmonary pathology, including congestion, pronounced vasodilation, hemorrhage, serum protein exudation, and leukocyte infiltration [38,39]. In our study, histopathological blood vessels and lungs suggest that the *P. multocida* infection caused pulmonary hemorrhage, infiltration of inflammatory cells, and damage to the intima of the blood vessels. This demonstrated that the damage to the blood vessels was another mechanism that needs to be particularly focused on to explain how *P. multocida* causes pneumonia.

Chinese herbal extracts and medicine show therapeutic potential against bacterial infections in veterinary practice [40]. Baicalin is a flavonoid compound with marked anti-inflammatory activity. Studies demonstrate that it effectively alleviates inflammatory injuries in both pulmonary and intestinal tissues and modulates gut microbiota balance in various disease models by suppressing key inflammatory pathways, including TLR4/NF-κB, PARP1/NF-κB/NLRP3, and PI3K/AKT/mTOR [41,42,43]. Moreover, prior investigation in our laboratory demonstrated that baicalin alleviated inflammatory responses in 3D4/21 cells, a porcine alveolar macrophage cell line, suggesting its potential therapeutic application for pulmonary inflammation [44]. The histological examination in this study further demonstrated that baicalin ameliorated pulmonary and vascular damage induced by *P. multocida*. Following baicalin treatment, the pulmonary and vascular structures were largely restored, characterized by well-defined alveolar contours, progressive normalization of elastic fibers within the vascular tissue, reduced edema in the lungs and vasculature, and diminished inflammatory cell infiltration. However, the absence of cytokine quantification (e.g., via ELISA or qPCR) in this study represents a limitation, and our subsequent work will integrate these quantitative endpoints to provide a more comprehensive mechanistic understanding. Furthermore, while this proof-of-concept study focused on establishing intrinsic efficacy, a direct comparison with standard antibiotics or NSAIDs is a crucial next step to properly position our intervention. These acknowledged limitations clearly define the scope of our current work and provide a clear roadmap for future validation and translation.

The NLRP3 inflammasome is the most extensively studied inflammasome and serves as a primary mediator of inflammatory responses, which plays a crucial role in the host’s defense against bacterial infections and tissue damage [45]. NLRP3 responds to various stimuli and forms an inflammasome complex that can cleave the precursors of IL-1β and IL-18 and then secrete mature IL-1β and IL-18 [46,47]. Studies have shown that high expression of NLRP3 has been observed in the development of various inflammatory responses caused by *P. multocida* [48,49]. Our study demonstrated that *P. multocida* infection increased the protein expression levels of NLRP3, IL-1β, and IL-18 in lung and vascular tissues, and baicalin alleviated the increase. This not only proves that the NLRP3 inflammasome and its signaling pathway play an important role in *P. multocida* infection but also illustrates the anti-inflammatory effect of baicalin, suggesting that baicalin may exert its anti-inflammatory effect through NLRP3 in *P. multocida*-induced pneumonia and vascular inflammatory injury, combined with the results of molecular docking.

The common clinical treatment for *P. multocida* infection is the combination of antibiotics and non-steroidal anti-inflammatory drugs, which can inhibit the enzymatic activity of cyclooxygenase (COX), especially COX-2, to alleviate the inflammatory response of the body [50,51]. This study found that the expression level of COX-2 increased in *P. multocida*-infected mice, while baicalin reduced its expression, suggesting that COX-2 may be another key protein for binding in molecular docking.

Interestingly, Hua et al. have reported that COX-2 can regulate the activation of NLRP3 inflammasome [52], while Tang et al. showed that the NLRP3 inflammasome can also up-regulate COX-2 through IL-1β [53], which indicates that the regulation between COX-2 and NLRP3 is a complex network. However, they are all important nodal proteins in the process of inflammatory response, and simultaneously targeted inhibition of COX-2 and NLRP3 can effectively block this signaling axis and significantly reduce the level of pro-inflammatory factors, such as IL-1β, thereby reducing inflammation. Furthermore, the selective COX-2 inhibitor, compound 6 k, not only suppresses COX-2 enzyme activity but also effectively inhibits the NF-κB/NLRP3 signaling pathway, leading to reduced expression of pro-inflammatory factors, which demonstrated a synergistic interaction between the NLRP3 inflammasome and COX-2 in the inflammatory process [54]. Therefore, based on the molecular docking results, the present study hypothesized that baicalin, as a lead compound, may play a role in mitigating *P. multocida*-induced pneumonia and vascular inflammatory injury by inhibiting the dual targets COX-2 and NLRP3. The mechanism and action site still need to be further studied.

## 5. Conclusions

In summary, in a mouse model of *P. multocida* infection, baicalin significantly reduced *P. multocida*-induced inflammatory injury in the lungs and blood vessels and decreased the protein expression of NLRP3, COX-2, IL-1β, and IL-18 in lung and blood vessel tissues. The signal inhibition of NLRP3 and COX-2 may be a key therapeutic pathway to treat *P. multocida*-induced pulmonary and vascular inflammatory injury. The data presented in this study should be available for preclinical and clinical studies evaluating the potential of baicalin to prevent inflammatory injury in the lungs and blood vessels caused by *P. multocida* infection.

## Data Availability

All data in this article are presented in the article.

## Abbreviations

AF3	AlphaFold3
COX	cyclooxygenase
DMSO	dimethyl sulfoxide
GLY	glycine
HIS	histidine
ILE	isoleucine
IL-1β	interleukin-1 beta
IL-18	interleukin-18
LYS	lysine
NLRP3	NOD, LRR, and pyrin domain-containing protein 3
PBS	phosphate-buffered saline
*P. multocida*	*Pasteurella multocida*
THR	threonine
VAL	valine

## References

[1] El-Demerdash A.S., Mowafy R.E., Fahmy H.A., Matter A.A., Samir M. (2023). Pathognomonic features of isolates among various avian species in Sharkia Governorate, Egypt. World J. Microb. Biot..

[2] Sahoo M., Baloni S., Thakor J.C., Kumar P., Thomas P., Nagaleekar V.K., Dhama K., Singh R., Singh K.P., Mani S. (2023). Pathology, virulence-associated gene profiling, antimicrobial susceptibility, and pathogenicity of untypeable capsular serotypes of *Pasteurella multocida* isolated from slaughtered pigs of India. Lett. Appl. Microbiol..

[3] Choi Y.K., Goyal S.M., Joo H.S. (2003). Retrospective analysis of etiologic agents associated with respiratory diseases in pigs. Can. Vet. J..

[4] Sun Q., Yu X.X., He D.X., Ku X.G., Hong B., Zeng W., Zhang H.F., He Q.G. (2022). Investigation and analysis of etiology associated with porcine respiratory disease complex in China from 2017 to 2021. Front. Vet. Sci..

[5] Almoheer R., Abd Wahid M.E., Zakaria H.A., Jonet M.A.B., Al-shaibani M.M., Al-Gheethi A., Addis S.N.K. (2022). Spatial, temporal, and demographic patterns in the prevalence of hemorrhagic septicemia in 41 countries in 2005–2019: A systematic analysis with special focus on the potential development of a new-generation vaccine. Vaccines.

[6] Yihunie F.B., Syoum A., Dubie T. (2025). Seroprevalence of *Pasteurella multocida* Serotype A2 in Cattle, Afar Region, Ethiopia. Vet. Med. Int..

[7] El-Tarabili R.M., Enany M.E., Alenzi A.M., Almessiry B.K., Alghamdi S., Kabrah A., Ghobashy M.O.I., Abdelrahman N.A., Youssef F.M., Algammal A.M. (2025). Unveiling resistance patterns, kmt1 sequence analyses, virulence traits, and antibiotic resistance genes of multidrug-resistant *Pasteurella multocida* retrieved from poultry and rabbits. Sci. Rep..

[8] Ali S., Tariq M.H.A., Yaqoob M., Haq M.U., Zahra R. (2025). Molecular epidemiology and characterization of antibiotic resistance of *Pasteurella multocida* isolated from livestock population of Punjab, Pakistan. Int. J. Vet. Sci. Med..

[9] Barsi Z.E., Allen J., Meza A. (2023). More than a case of cellulitis: *Pasteurella multocida* bacteremia. Cureus.

[10] He J., Yang Z., Wang M., Jia R., Chen S., Liu M., Zhao X., Yang Q., Wu Y., Zhang S. (2024). Integrative and conjugative elements of *Pasteurella multocida*: Prevalence and signatures in population evolution. Virulence.

[11] Akcakavak G., Karatas O., Tuzcu N., Tuzcu M. (2024). Determination of apoptosis, necroptosis and autophagy markers by real-time PCR in naturally infected *Pneumonic pasteurellosis* caused by *Pasteurella multocida* and *Mannheimia haemolytica* in cattle. Pak. Vet. J..

[12] Wang J.P., Luo J.Q., Rotili D., Mai A., Steegborn C., Xu S.W., Jin Z.G. (2024). SIRT6 protects against lipopolysaccharide-induced inflammation in human pulmonary lung microvascular endothelial cells. Inflammation.

[13] de Oliveira J.X., Morés M.A.Z., Rebellato R., Kich J.D., Cantao M.E., Klein C.S., Guedes R.M.C., Coldebella A., de Barcellos D.E.S.N., Morés N. (2018). Pathogenic variability among type A isolates from Brazilian pig farms. BMC Vet. Res..

[14] Lin L., Bi H., Yang J., Shang Y., Lv Q., Zhang D., Huang X., Zhao M., Wang F., Hua L. (2024). *Pasteurella multocida* infection induces blood-brain barrier disruption by decreasing tight junctions and adherens junctions between neighbored brain microvascular endothelial cells. Vet. Res..

[15] Smallman T.R., Perlaza-Jimenez L., Wang X., Korman T.M., Kotsanas D., Gibson J.S., Turni C., Harper M., Boyce J.D. (2024). Pathogenomic analysis and characterization of *Pasteurella multocida* strains recovered from human infections. Microbiol. Spectr..

[16] Li S.L., Gu Q.B., Li B.R., Abi K., Yang F.L. (2025). High prevalence of virulence genes and multi-drug resistance in *Pasteurella multocida* from goats in Sichuan, China. Vet. J..

[17] Holschbach C.L., Raabis S.M., Ollivett T.L. (2019). Effect of antibiotic treatment in preweaned Holstein calves after experimental bacterial challenge with *Pasteurella multocida*. J. Dairy Sci..

[18] Sun Y., Mao W., Cao J.S., Hao P.G., Su J.G., Yin K.W., Gu K.R., Zhao H.X. (2024). Chinese medicine monomers inhibit biofilm formation in multidrug-resistant P. isolated from cattle respiratory infections. Pak. Vet. J..

[19] Guo P.L., Zeng M.N., Liu M., Zhang Y.H., Jia J.F., Zhang Z.Y., Liang S.L., Zheng X.K., Feng W.S. (2025). Zingibroside R1 isolated from achyranthes bidentata blume ameliorates LPS/D-GalN-induced liver injury by regulating succinic acid metabolism via the gut microbiota. Phytother. Res..

[20] Chen W., Zhang H., Wang J.F., Hu X.J. (2019). Flavonoid Glycosides from the bulbs of Lilium speciosum var. gloriosoides and their potential antiviral activity against RSV. Chem. Nat. Compd..

[21] Wei Q., Yu Z.P., Yang P., Chen X.H. (2024). Baicalin maintains articular cartilage homeostasis and alleviates osteoarthritis by activating FOXO1. J. Med. Food.

[22] Lin M.Y., Cheng W.T., Cheng H.C., Chou W.C., Chen H.I., Ou H.C., Tsai K.L. (2021). Baicalin enhances chemosensitivity to doxorubicin in breast cancer cells via upregulation of oxidative stress-mediated mitochondria-dependent apoptosis. Antioxidants.

[23] Li K.W., Liang Y.Y., Cheng A., Wang Q., Li Y., Wei H.C., Zhou C.Z., Wan X.H. (2021). Antiviral properties of baicalin: A concise review. Rev. Bras. Farmacogn..

[24] Liu Y.F., Jiang C.X., Peng L., Li Z.M., Wang J.T., Liao X.W., Guo W.Y. (2023). Discovery of metal complexes with antibacterial properties in aqueous extracts of and a study of the antibacterial properties of the baicalin-manganese complex. Inorg. Chem. Front..

[25] Jiang J.J., Kao T.C., Hu S.H., Li Y.B., Feng W.Y., Guo X.C., Zeng J.H., Ma X. (2023). Protective role of baicalin in the dynamic progression of lung injury to idiopathic pulmonary fibrosis: A meta-analysis. Phytomedicine.

[26] Wang D.N., Li Y. (2023). Pharmacological effects of baicalin in lung diseases. Front. Pharmacol..

[27] Wang S.M., Wu M.Y., Ding J.R., Tan W., Jiang H.B. (2024). Baicalin alleviates acute lung injury in vivo and in vitro. Int. Immunopharmacol..

[28] Sharawi Z.W., Ibrahim I.M., Abd-alhameed E.K., Althagafy H.S., Jaber F.A., Harakeh S., Hassanein E.H.M. (2024). Baicalin and lung diseases. Naunyn-Schmiedeberg’s Arch. Pharmacol..

[29] Shen B.Y., Zhang H.Q., Zhu Z.J., Ling Z.X., Zeng F.Y., Wang Y.Z., Wang J.G. (2023). Baicalin relieves LPS-induced lung inflammation via the NF-κB and MAPK pathways. Molecules.

[30] Ding X.M., Pan L., Wang Y., Xu Q.Z. (2016). Baicalin exerts protective effects against lipopolysaccharide-induced acute lung injury by regulating the crosstalk between the CX3CL1-CX3CR1 axis and NF-κB pathway in CX3CL1-knockout mice. Int. J. Mol. Med..

[31] Li L., Cui H.R., Zhang Y., Xie W., Lin Y., Guo Y.F., Huang T.X., Xue B., Guo W.B., Huang Z.F. (2023). Baicalin ameliorates multidrug-resistant induced pulmonary inflammation in rat via arginine biosynthesis. Biomed. Pharmacother..

[32] Zhao G.F., Tang Y.H., Dan R.T., Xie M.H., Zhang T.C., Li P., He F., Li N.Z., Peng Y.Y. (2024). *Pasteurella multocida* activates apoptosis via the FAK-AKT-FOXO1 axis to cause pulmonary integrity loss, bacteremia, and eventually a cytokine storm. Vet. Res..

[33] Lu Q.R., Wang L.Y., Jiang X.P., Han W.T., Guo P., Liu Y., Fu S.L., Xiong J.L., Wu Z.Y., Qiu Y.S. (2025). Protective effects of 18β-glycyrrhetinic acid on *Pasteurella multocida*-induced vascular inflammatory injury in mice. Front. Vet. Sci..

[34] Guo P., Lu Q.R., Hu S.Y., Yang Y.Q., Wang X.R., Yang X.Z., Wang X. (2023). Daucosterol confers protection against T-2 toxin induced blood-brain barrier toxicity through the PGC-1α-mediated defensive response and in vivo. J. Hazard. Mater..

[35] Lu Q.R., Hu S.Y., Guo P., Zhu X.H., Ren Z.C., Wu Q.H., Wang X. (2021). PPAR-γ with its anti-fibrotic action could serve as an effective therapeutic target in T-2 toxin-induced cardiac fibrosis of rats. Food Chem. Toxicol..

[36] Li M.M., Wang S.Y., Dong H.Y., Wang M., Sun Y., Bi Y., Chen L.X., Naseem A., Jiang H., Li H. (2025). Harnessing natural products for myocardial infarction therapy: Mechanistic insights and translational opportunities. Pharmacol. Res..

[37] Fu Q.Y., Jiang J.M., Li X.B., Zhai Z., Wang X.M., Li C.R., Chen Q.L., Man C.R.G., Du L., Wang F.Y. (2023). Activation of MyD88-dependent TLR signaling modulates immune response of the mouse heart during infection. Microorganisms.

[38] Wu Q.C., Yu L.J., Qiu J.M., Shen B.Y., Wang D., Soromou L.W., Feng H.H. (2014). Linalool attenuates lung inflammation induced by *Pasteurella multocida* via activating Nrf-2 signaling pathway. Int. Immunopharmacol..

[39] Cheng Y., Wang K.C., Lin L.S., Zhao X.K., Pan Z.H., Zhou Z.L. (2020). Differences in pathogenicity and virulence-associated gene expression among strains with high and low virulence in a lung tissue model. Microb. Pathog..

[40] Xu F.B., Yao Y.C., Li Y.F., Wang W.M., Wu Z.Y. (2024). A review on the application of traditional to modern approaches of chinese herbal veterinary medicines: Current status and challenges. J. Food Biochem..

[41] Long Y., Li X.Q., He X.F., Wang Z.S., Wu Y.Y., Sun L.M., Ma Y., Deng J., Hu Y., Li N. (2025). Baicalin liposomes ameliorate cerebral ischemia-reperfusion-induced acute lung injury by modulating the inflammatory response. Brain Res..

[42] Song D., Wei W.F., Zhang J., Zhang L., Huo J.H., Wang W.M. (2025). The mechanism of baicalin in improving pulmonary inflammatory response and injury and regulating intestinal flora in Mycoplasma pneumoniae pneumonia mice. Cell. Signal..

[43] Lu Q.R., Wang N., Wen D.F., Guo P., Liu Y., Fu S.L., Ye C., Wu Z.Y., Qiu Y.S. (2024). Baicalin attenuates lipopolysaccharide-induced intestinal inflammatory injury via suppressing PARP1-mediated NF-κB and NLRP3 signalling pathway. Toxicon.

[44] Zong B.B., Xiao Y., Ren M.X., Wang P.Y., Fu S.L., Qiu Y.S. (2023). Baicalin Weakens the Porcine ExPEC-Induced Inflammatory Response in 3D4/21 Cells by Inhibiting the Expression of NF-κB/MAPK Signaling Pathways and Reducing NLRP3 Inflammasome Activation. Microorganisms.

[45] Ramachandran R., Manan A., Kim J., Choi S. (2024). NLRP3 inflammasome: A key player in the pathogenesis of life-style disorders. Exp. Mol. Med..

[46] Grebe A., Hoss F., Latz E. (2018). NLRP3 Inflammasome and the IL-1 Pathway in Atherosclerosis. Circ. Res..

[47] Sendler M., van den Brandt C., Glaubitz J., Wilden A., Golchert J., Weiss F.U., Homuth G., Chama L.L.D., Mishra N., Mahajan U.M. (2020). NLRP3 inflammasome regulates development of systemic inflammatory response and compensatory anti-inflammatory response syndromes in mice with acute pancreatitis. Gastroenterology.

[48] Fang R.D., Du H.H., Lei G.H., Liu Y.J., Feng S.W., Ye C., Li N.Z., Peng Y.Y. (2019). NLRP3 inflammasome plays an important role in caspase-1 activation and IL-1β secretion in macrophages infected with. Vet. Microbiol..

[49] Ran J.R., Yin H., Xu Y.T., Wang Y., Li G., Wu X.P., Peng L.C., Peng Y.Y., Fang R.D. (2023). RACK1 mediates NLRP3 inflammasome activation during infection. Vet. Res..

[50] Prakoso Y.A., Susilo A., Widyarini S. (2024). The standardization and efficacy of fermented *Crescentia cujete* (L.) in combination with enrofloxacin against artificially induced *Pneumonic pasteurellosis* in rat models. Open Vet. J..

[51] Giacominelli-Stuffler R., Marruchella G., Storelli M.M., Sabatucci A., Angelucci C.B., Maccarrone M. (2012). 5-Lipoxygenase and cyclooxygenase-2 in the lungs of pigs naturally affected by enzootic pneumonia and porcine pleuropneumonia. Res. Vet. Sci..

[52] Hua K.F., Chou J.C., Ka S.M., Tasi Y.L., Chen A., Wu S.H., Chiu H.W., Wong W.T., Wang Y.F., Tsai C.L. (2015). Cyclooxygenase-2 Regulates NLRP3 Inflammasome-Derived IL-1β Production. J. Cell. Physiol..

[53] Tang B., Liu D., Chen L.Y., Liu Y. (2020). NLRP3 inflammasome inhibitor MCC950 attenuates primary dysmenorrhea in mice via the NF-κB/COX-2/PG pathway. J. Inflamm..

[54] Ju Z.R., Xu J.D., Tang K.S., Chen F.E. (2024). Structural modification based on the diclofenac scaffold: Achieving reduced colitis side effects through COX-2/NLRP3 selective inhibition. Eur. J. Med. Chem..

